# Relationship of treatment satisfaction to medication adherence: findings from a cross-sectional survey among hypertensive patients in Palestine

**DOI:** 10.1186/1477-7525-11-191

**Published:** 2013-11-06

**Authors:** Sa’ed H Zyoud, Samah W Al-Jabi, Waleed M Sweileh, Donald E Morisky

**Affiliations:** 1 Poison Control and Drug Information Center (PCDIC), College of Medicine and Health Sciences, An-Najah National University, Nablus, Palestine; 2 Department of Pharmacology and Toxicology, College of Medicine and Health Sciences, An-Najah National University, Nablus, Palestine; 3 WHO Collaborating Centre for Drug Information, National Poison Centre, Universiti Sains Malaysia (USM), Penang, Malaysia; 4 Department of Clinical Pharmacy and Pharmacotherapy, College of Medicine and Health Sciences, An-Najah National University, Nablus, Palestine; 5 Department of Community Health Sciences UCLA School of Public Health, Los Angeles, CA, USA

**Keywords:** TSQM, Treatment satisfaction, Medication adherence, Hypertension, MMAS

## Abstract

**Background:**

The concepts of medication adherence and Treatment satisfactions are commonly used in clinical research for assessing pharmaceutical care and improving treatment outcomes. Generally, one would expect a positive relationship between the two concepts. The objectives of this study were to investigate the factors associated with adherence to antihypertensive therapy among hypertensive patients and to assess the relationship between antihypertensive medication adherence and treatment satisfaction.

**Methods:**

A cross-sectional study was conducted, adopting the Morisky eight-item Medication Adherence Scale (MMAS) for the assessment of medication adherence and using the Treatment Satisfaction Questionnaire for Medication (TSQM 1.4) for the assessment of treatment satisfaction. Descriptive and comparative statistics were used to describe socio-demographic and disease-related characteristics of the patients. All analyses were performed using SPSS v 15.0.

**Results:**

Four hundred and ten hypertensive patients were enrolled in the study. The mean age of participants was 58.38 ± 10.65 years; 52% were female and 36.8% had low antihypertensive medication adherence. There was a significant difference in the mean scores in the Effectiveness (p < 0.001), Convenience (p < 0.001), and Global Satisfaction (p < 0.001) domains, but not in the Side Effects (p = 0.466) domain among patients with different levels of adherence. After adjustment for covariates using multiple linear regression, global treatment satisfaction was still statistically significantly (p = 0.001) associated with medication adherence.

**Conclusions:**

Low treatment satisfaction may be an important barrier for achieving high rates of adherence to treatment. These study findings could be helpful in clinical practice, mainly in the early treatment of hypertensive patients, at a point where improving treatment satisfaction is still possible.

## Background

Hypertension is a condition with a tremendous economic and public health impact which contributes to disability, health care costs and mortality [1]. Although the management and control of hypertension reduces morbidity and mortality, the percentage of patients with controlled hypertension has been reported to vary between 5.4% and 58% worldwide [2]. One factor contributing to less than ideal blood pressure control is low adherence to treatment [3,4]. Multiple factors that influence patient adherence to prescribed therapies have been illustrated; these include treatment satisfaction, high incidence of adverse effects of blood pressure-lowering drugs, health care system issues, quality of life, socio-demographics, lack of knowledge regarding hypertension, and treatment and clinical variables [5-7]. Treatment satisfaction is believed to affect the patient’s health- related decision making [8]. The impact of low treatment satisfaction on medication adherence is of particular alarm in patients with chronic diseases. It has been found that up to one-half of patients with chronic illness end up making medication-related decisions without looking for medical advice, becoming “non-adherent” to such an extent that they compromise the effectiveness of treatment [8]. Therefore, health care providers need to identify patients’ level of satisfaction with the medications they are using. To evaluate this, measures for assessing treatment satisfaction have been developed [9].

Despite the assessment of adherence, treatment satisfaction, or both in many studies, only limited studies have been performed to measure the relationship between adherence to drug treatment and treatment satisfaction among hypertensive patients. Given the small number of studies in which the association between adherence and treatment satisfaction has been investigated [6,10,11], a review of the literature revealed no studies on medication adherence and treatment satisfaction among patients with hypertension in the Arab world. This is important given that culture and religion might play a role in health-related issues; the objectives of this study were to investigate the factors associated with adherence to antihypertensive therapy among hypertensive patients and to assess the relationship between antihypertensive medication adherence and treatment satisfaction.

## Methods

### Study design, participants and setting

A cross-sectional study design was used to address the research goals. The study was conducted in outpatients’ clinics at Al-Makhfyah primary health care clinic and at Alwatani Hospital, Nablus district in Palestine. These are the largest public institutes in Nablus district. Being generalised in nature, they provide health care for all chronic illnesses. Most patients in Palestine consult within the government health care system [12].

In two population-based cross-sectional studies carried out previously, the rate of hypertension in two communities from the West Bank ranged from 21.5% to 25.4% in adults aged 30–65 years [13]. Therefore, a prevalence-based sample of 385 hypertensive patients was selected for this study [14]. In order to minimise erroneous results and increase the study reliability, the target sample size was increased to 410 patients. Therefore, a convenience sample of 410 hypertension outpatients was identified between July 2012 and October 2012.

The inclusion criteria were (1) patients diagnosed with hypertension at least six months before recruitment into the study, (2) patients treated for hypertension with antihypertensive medications, (3) aged 18 years and over, (4) able to recognise their medications from the total medications that they took daily, and (5) patients who were willing to participate and who had given verbal consent to participate in the study.

### Ethical approval

This study received approval from the Palestinian Ministry of Health and Institutional Review Board (IRB) at An-Najah National University. Verbal consent was also obtained from the patients prior to the commencement of the study.The IRB considered waiving the requirement to obtain verbal consent for our protocols that were clearly below minimal risk and the research did not involve any therapeutic intervention.

### Data collection

On the day of the assessment, data were collected using a set of questionnaires. Four main variables were used: (1) socio-demographic data, (2) clinical hypertension related data, (3) medication adherence profile of hypertensive patients; and (4) treatment satisfaction profile of hypertensive patients.

### Assessment of medication adherence

The Morisky scale, also known as the Morisky Medication Adherence Scale (MMAS), was used for the assessment of medication adherence [3,15]. The English version of the MMAS was translated into Arabic and was approved by Professor Morisky through e-mail communications [16]. The Arabic version of the MMAS is an eight-item questionnaire with seven yes/no questions and one question answered on a five-point Likert scale. According to the scoring system of the MMAS, scores can range from zero to eight, with MMAS scores of <6, 6 to <8 and 8 reflecting low, medium and high adherence, respectively [3,17]. The internal consistency and validity of the questionnaire was ensured (Cronbach’s alpha value was 0.723 for the instrument used in our study).

### Assessment of treatment satisfaction

Treatment satisfaction was tested using the Arabic version of the Treatment Satisfaction Questionnaire for Medication (TSQM 1.4), which the researchers obtained from Quintiles Strategic Research Services. The TSQM 1.4 is a 14-item psychometrically robust and validated instrument comprising four domains [9]. Effectiveness (questions 1–3), Side Effects (questions 4–8), Convenience (questions 9–11), and Global Satisfaction (questions 12–14). The TSQM 1.4 domain scores were calculated as recommended by the instrument’s authors and described in detail elsewhere [6,18]. TSQM 1.4 domain scores range from 0 to 100, with higher scores representing higher satisfaction in that domain. The “Global Satisfaction” scale of the TSQM is used to assess the overall level of satisfaction or dissatisfaction with medications that patients are taking. Patients’ global rating of dissatisfaction with treatment is the strongest predictor of non-adherence and non-persistence with medication use, thereby affecting the clinical effectiveness and efficiency of medical care. Patients who recognize their medication to be ineffective, laden with side effects, very inconvenient, or as having more negative than positive characteristics are less likely taking their medications as prescribed. This in turn can impact the effectiveness of treatment and may result in service inefficiencies associated with treatment failure. TSQM provides a unique opportunity to compare various medications used to treat a particular illness on the three primary dimensions of treatment satisfaction (Effectiveness, Side Effects, Convenience), as well as patients’ overall rating of Global Satisfaction based on the relative importance of these primary dimensions to patients. Routine assessment of patients’ level of treatment satisfaction provides a way for health care providers to screen individuals whose current medication experiences may increase the risk of poor medication adherence and persistence. If collected from many patients, such information could promote a deeper consideration of patients’ perspectives when evaluating drawbacks of various treatment strategies [6,9,18]. The internal consistency and validity of the questionnaire was ensured for the instrument used in our study namely Effectiveness (3 items, Cronbach’s Alpha =0.92), Side effects (4 items, Cronbach’s Alpha =0.97), Convenience (3 items, Cronbach’s Alpha =0.86), and Global Satisfaction scale (3 items, Cronbach’s Alpha =0.89).

### Data collection procedure

Data collection was carried out by face-to-face interviews with the patients by trained senior Pharm D students. The data collection was pre-tested in through a pilot study of 10 patients who were not included in the final analysis to check for the understandability and language clarity of questions, and all valid comments were taken into consideration by the principal researchers in the main survey, and the modified version was reviewed by experts to ensure content and construct validity. A total of 410 patients were eligible and included in the final analysis with a response rate of 96.4%. Regular evaluations took place throughout the abstraction period to identify any problems in data collection, interpretation of definitions, and application of study criteria. Before commencing data analysis, an extensive series of checks were performed for data consistency, proper sequences of data, and an evaluation of missing or incomplete data.

### Statistical analysis

Data were entered and analysed using the Statistical Package for Social Sciences (SPSS; SPSS Inc., Chicago, IL, USA) program version 15. Spearman’s correlation coefficient was used to identify the relationship between the MMAS score and TSQM scores. Multiple linear regression analysis was used to evaluate the correlation of the factors with medication adherence. Internal consistency was assessed using Cronbach’s alpha. Descriptive and comparative statistics were used to describe socio-demographic and disease-related characteristics of the patients. The internal consistency and validity of the MMAS and TSQM were ensured. Variables were tested for normality using the Kolmogorov–Smirnov test. Intergroup differences in MMAS scores were assessed for statistical significance using either Mann–Whitney or Kruskal–Wallis test for numerical data, as appropriate. Furthermore, differences in means among MMAS levels and TSQM scores were tested using one-way ANOVA with Tukey’s post hoc test. The significance level was set at P < 0.05.

## Results

### Demographic characteristics

Table 1 describes the socio-demographic characteristics of the study participants. Two hundred and thirteen (52%) patients of the study population were female. The mean age of the study population was 58.38 ± 10.65 years (range: 30–90 years). The majority (46.2%) of patients in the study population were obese. A total of 245 patients (59.8%) had low income, 194 (47.3%) had village residency and the majority of patients had a primary level of education. The mean duration of hypertension was 7.34 years (SD = 5.90). The median number of total medications per day was 4.0 (interquartile range: 3.0–6.0). The majority of patients (86.8%) were on combination antihypertensive medicines.

**Table 1:** Socio-demographic and disease-related characteristics of the study patients with differences in adherence total scores (N = 410)

**Variable**	**Frequency (%)**	**Morisky score**	***p*-value**
		**Median**	
	**N = 410**	**[interquartile range]**	
**Age category**			
28-37	13 (3.2)	8.0 [5.3-8.0]	0.042^a^
38-47	39 (9.5)	7.0 [4.8-8.0]	
48-57	149 (36.3)	7.0 [4.8-8.0]	
58-67	119 (29)	7.0 [5.0-8.0]	
≥68	90 (22)	5.8 [4.7-7.8]	
**Gender**			
Male	197 (48.0)	6.8 [4.8-8.0]	0.048^b^
Female	213 (52.0)	7.0 [5.5-8.0]	
**BMI**			
Normal	35 (8.5)	7.0 [5.3-8.0]	0.231^a^
Overweight	183 (44.6)	7.0 [4.8-8.0]	
Obese	192 (46.2	6.8 [4.8-8.0]	
**Residency**			
Village	194 (47.3)	7.0 [4.8-8.0]	0.626^a^
City	166 (40.5)	6.8 [4.8-8.0]	
Palestinian refugee camps	50 (12.2)	7.6 [5.0-8.0]	
**Occupation**			
Employed	156 (38.0)	7.0 [4.8-8.0]	0.089^a^
Unemployed	63 (15.4)	6.0 [4.8-8.0]	
Housewife	191 (46.6)	7.0 [5.0-8.0]	
**Marital status**			
Single	13 (3.2)	8.0 [6.5-8.0]	0.012^a^
Married	346 (84.4)	7.0 [4.8-8.0]	
Divorced	12 (2.9)	5.1 [3.0-6.8]	
Widowed	39 (9.5)	6.8 [4.5-8.0]	
**Income (NIS**^**c**^**)**			
Less than 2000	245 (59.8)	7.0 [4.8-8.0]	0.101^a^
2000-5000	150 (36.6)	7.0 [4.8-8.0]	
More than 5000	15 (3.7)	6.5 [4.5-8.0]	
**Education**			
No formal	40 (9.8)	6.8 [4.5-8.0]	0.009^a^
Primary	149 (36.3)	7.0 [5.0-8.0]	
Secondary	112 (27.3)	6.5 [4.8-8.0]	
University	109 (26.9)	7.8 [5.8-8.0]	
**Duration of the disease**			
< 1 year	4 (1.0)	7.3 [3.7-7.9]	0.451^a^
1-3 years	120 (29.3)	7.0 [4.8-8.0]	
4-5 years	93 (22.7)	7.0 [5.0-8.0]	
≥ 5 years	193 (47.1)	6.8 [4.8-8.0]	
**Therapy type**			
Mono therapy	54 (13.2)	8.0 [5.5-8.0]	0.004^b^
Multi therapy	356 (86.8)	6.8 [4.8-8.0]	
**Total number of chronic diseases**			
0	151 (36.8)	7.0 [5.0-8.0]	0.016^a^
1	159(38.8)	6.8 [4.8-8.0]	
2	79 (19.3)	7.0 [5.5-8.0]	
≥4	21 (5.1)	4.8 [3.6-7.4]	
**Total number of medications**			
1-3	166(40.5)	7.3 [5.0-8.0]	0.006^a^
4-6	172(42.0)	6.8 [4.8-8.0]	
≥7	72(17.6)	5.9 [4.7-7.8]	

*Abbreviations*: *BMI* Body mass index; *NIS* New Israeli Shekel.

^a^Statistical significance of differences calculated with the Kruskal-Wallis test.

^b^Statistical significance of differences calculated with the Mann–Whitney U test.

^c^1 Israeli New Sheqel equals 0.27 US Dollar.

### Medication adherence

The median MMAS score was 7.0, (interquartile range: 4.8–8.0). Using published MMAS-8 thresholds [3], 36.8% of participants had low, 26.8% had medium and 36.2% had high antihypertensive medication adherence. As shown in Table 1, a significant difference in MMAS score was found between participant groups according to age, marital status, educational level, total number of chronic diseases, and total number of medications (Kruskal–Wallis test; p < 0.05), as well as gender and therapy type (Mann–Whitney test, p < 0.05). Patients aged over 68 years had a lower median MMAS score than those age groups of less than 68 years. Furthermore, female gender was associated with a higher MMAS score. The study found that MMAS score increased as the educational level of patients increased. Also, it was found that MMAS score decreased as the total number of chronic diseases and total number of medications increased. A significantly lower MMAS score was seen in participants receiving combination therapy as hypertension treatment.

### Treatment satisfaction and its relationship between medication adherence

TSQM score was measured as good. The average satisfaction scores in the Effectiveness, Side Effects, Convenience, and Global Satisfaction domains were 62.1 ± 20.5, 86.0 ± 26.5, 67.2 ± 14.0, and 72.1 ± 23.1, respectively. Furthermore, the median Effectiveness score was 66, (interquartile range: 50–77), the median Side Effects score was 100, (interquartile range: 98–100), the median Convenience score was 66, (interquartile range: 61–78), and the median Global Satisfaction score was 83, (interquartile range: 58–92). There were a significantly strong positive correlation between Global Satisfaction score and Effectiveness domain (*r* = 0.79; p < 0.001), Side Effects domain (*r* = 0.24; p < 0.001), and Effectiveness (*r* = 0.59; p < 0.001).

There was a significant difference in the mean scores in the Effectiveness (p <0.001), Convenience (p < 0.001), and Global Satisfaction (p <0.001) domains, but not in the Side Effects (p = 0.466) domain among patients with different levels of adherence. Patients with a high adherence rate had the highest global treatment satisfaction scores compared with those with a low or medium adherence rate. The Spearman’s rank order correlation coefficient between total adherence and overall TSQM score indicated a significant positive correlation (*r* = 0.337; p < 0.001).

### Multiple linear regression analysis

The previous analysis did not take into account the effect of confounding factors, which may affect the relationship between these factors and medication adherence in patients with hypertension. From the univariate analysis, age, gender, marital status, educational level, therapy type, number of chronic diseases and amount of medication were found to be statistically significant P < 0.05 (Table 1). As recommended by Tabachnick and Fidell [19], the independent variables should be strongly related to the dependent variable but they should not strongly related to each other. In the current study, because there were inter-correlations between the domains of TSQM, highly inter-correlated variables were excluded. Therefore, the general global treatment satisfaction domain was included in the multiple linear regression model, while other domains were excluded (Table 2). After adjusting the covariates using multiple regression, global treatment satisfaction was still statistically significantly (p = 0.001) associated with medication adherence (R = 0.373; adjusted R^2^ = 0.122; F = 8.107; df = 8; p < 0.001). No multicollinearity was found among the independent variables (the minimum tolerance was 1.06 and the maximum variance inflation factor (VIF) for multicollinearity was 2.96). Table 2 shows the contribution of each independent variable to adherence. Beta values are usually used to achieve this purpose, with the highest absolute value indicating the strongest contribution to adherence. In our model the largest beta coefficient was 0.30, which was for global treatment satisfaction variable (Table 2). This means that global treatment satisfaction made the strongest unique contribution to explaining variations in medication adherence. Thus, higher scores in TSQM predicted higher scores in MMAS.

**Table 2:** Multiple linear regression analysis of association between factors and medication adherence

**Variable**	**Correlation coefficient**	**Regression coefficient (β)**	**95% CI for B**	***p*-value**
**Age**	0.03	0.04	-0.13-0.26	0.514
**Gender**	0.16	0.16	0.25-0.94	0.001
**Marital status**	-0.07	-0.08	-0.51-0.06	0.128
**Educational level**	0.02	0.02	-0.14-0.21	0.706
**Therapy type**	-0.06	-0.06	-0.87-0.21	0.234
**Number of chronic diseases**	-0.04	-0.07	-0.44-0.16	0.367
**Number of medications**	-0.01	-0.01	-0.43-0.37	0.875
**Global treatment satisfaction**	0.29	0.30	0.02-0.03	<0.000

*Abbreviations: β* Beta standardized linear regression coefficient; *95% CI* 95% confidence interval for B unstandardized linear coefficients.

## Discussion

To the best of our knowledge, no studies of the determinants of adherence and treatment satisfaction among patients with hypertension in Palestine have been carried out previously. Although limited studies have reported medication adherence among Arab populations suffering from different diseases, there is a lack of data concerning medication adherence and treatment satisfaction solely among hypertensive patients. This is therefore the first attempt to determine the relationship between medication adherence and treatment satisfaction in Palestine and Arab hypertensive patients. An improved understanding of the determinants associated with medication adherence and treatment satisfaction has become an important outcome in management strategies for hypertension [6,18]. Such understanding will help predicting hypertensive patient’s life and perceptions of illness. Therefore, the aim of this pioneering study in Palestinian health care settings was to assess the relationship between medication adherence and treatment satisfaction in hypertensive patients. Determining patient adherence to antihypertensive medications in outpatient settings is an important first step for physicians and other health care providers in understanding the effectiveness of or satisfaction to the treatments they prescribe, identifying barriers to treatment, and improving blood-pressure control [20]. It is important for health care providers to consider low treatment satisfaction or low medication adherence as a factor contributing to poor blood pressure control to actively engage patients in the selection of strategies to improve adherence [9,18,21]. The ability to identify indicators of low medication adherence is crucial for both improving clinical care and determining targets of intervention for the prevention of complications and treatment of hypertension.

A review of the literature revealed that adherence to treatment of hypertension is influenced by a number of factors [3,22-24]. Medication adherence rates have been shown to be related with age, gender, and race [25,26]. Other factors are modifiable which reported negatively impact adherence to prescribed medications include depression [27], lack of knowledge regarding hypertension and its treatment [28], complexity of medication regimen [29], side effects of medication [30], patient satisfaction [3], and poor quality of life [7]. In our study, we identified one modifiable variable in the multiple regression model that predict medication adherence. Some of the interesting findings in the model indicated that treatment satisfaction was significantly associated with medication adherence. This implies the need for effective communication between the physician or pharmacist and patients to improve understanding regarding hypertension and its treatment.

Treatment satisfaction may be associated with medication adherence for several reasons, including patients’ attitudes or beliefs towards taking antihypertensive medications [31]. Morisky et al. [3] and Bharmal et al. [6] stated that the exact mechanism through which treatment satisfaction is associated with medication adherence is unknown; however, low treatment satisfaction appears to be associated with psychosocial well-being which can negatively impact a patient’s ability to manage their chronic illnesses and other health problems. Previous studies performed among hypertensive patients have linked treatment satisfaction to numerous factors, which are recognised to be precursors to medication adherence. These include patients’ beliefs, their perceived level of competence, knowledge and attitudes about disease treatment, and their overall attitude to life [3,6,10,11]. Further research is needed to understand the real mechanisms through which treatment satisfaction is associated with adherence to antihypertensive medications.

Our study is the first one to assess adherence and satisfaction among Arab patients with hypertension using validated tools; however, our study has a few limitations. Firstly, the major limitation of this study is its cross-sectional design, which precludes causal relationships to be identified. Secondly, no account has been made of hypertension severity, therefore, a more accurate measure may be needed to assess whether blood pressure is well controlled or not. Finally, the participants were drawn from one district and therefore, our findings cannot be generalised to the entire country.

## Conclusions

We conclude that participants with low treatment satisfaction are more likely to have lower adherence to antihypertensive medications. In conclusion, the results of this study support the hypothesis that treatment satisfaction is reliable indicators of adherence to antihypertensive medications in patients presenting with hypertension in Palestine. Thus, low treatment satisfaction may be an important barrier to achieving high rates of adherence to treatment. Patients should be educated about the advantages of self-management of diseases, and the common perception that drugs are inherently unsafe has to be eliminated. Further research is also recommended in order to identify appropriate and targeted interventions in an effort to improve treatment satisfaction in patients with hypertension.

## Abbreviations

MMAS: Morisky eight-item medication adherence scale; TSQM: Treatment satisfaction questionnaire for medication; IRB: Institutional Review Board; SPSS: Statistical Package for Social Sciences; SD: Standard deviation; BMI: Body mass index; NIS: New Israeli Shekel; SE: Standard error; CI: Confidence interval.

## Competing interests

The authors declare that they have no competing interests.

## Authors’ contributions

All authors were involved in drafting the article and all authors approved the final version to be submitted for publication. SZ, SA and WS have added an intellectual significant value to the manuscript. DM contributed the adherence scale and concept of medication adherence. The project was initially and originally conceptualized and designed by (SZ, SA, WS). Data collection was made by the help of a group of senior Pharm D students who volunteered to help in data collection. The students were acknowledged for their voluntary help at the acknowledgement section. Project approval, data analysis, interpretation, manuscript writing, submission and revision were made by the research team (SZ, SA and WS).

## Authors’ information

Dr. Sa’ed Zyoud is an assistant professor of clinical toxicology/pharmacy and the head of a research group in the field of clinical toxicology, clinical pharmacology/pharmacy, social pharmacy, pharmacoepidemiology and drug safety. S.Z and the research group have published many articles in leading international and high reputation journals. The research group has also supervised many students in the fields of nursing, public health and pharmacy.
